# Comparative Evaluation of the Remineralization Potential of Fluoride-Free Bioactive Toners and Fluoride-Containing Functionalized Tricalcium Phosphate Toothpaste on the Artificially Demineralized Enamel: An *In Vitro* Study

**DOI:** 10.1055/s-0046-1822829

**Published:** 2026-05-27

**Authors:** Apa Juntavee, Niwut Juntavee, Ratha Rakchumchon, Supichaya Jitijesadaporn, Kochanipa Jungtanasombat, Rachata Somsub

**Affiliations:** 1 Department of Preventive Dentistry, Faculty of Dentistry, Khon Kaen University, Khon Kaen, Thailand; 2 Department of Prosthodontics, Faculty of Dentistry, Khon Kaen University, Khon Kaen, Thailand; 3 Division of Biomaterials Research, Faculty of Dentistry, Khon Kaen University, Khon Kaen, Thailand

**Keywords:** apacider, calcium phosphate-based remineralizing agent, fluoride, fluoride-free remineralizing agent, functionalized tricalcium phosphate, nanohydroxyapatite, remineralization

## Abstract

**Objectives:**

Management of demineralized enamel currently focuses on the use of remineralizing materials. This study evaluated the remineralizing efficacy of two fluoride-free bioactive toners—BAT-1 (nanohydroxyapatite, calcium phosphate, and antibacterial agents) and BAT-2 (calcium phosphate and antibacterial agents)—compared with fluoride-containing functionalized tricalcium phosphate toothpaste (CPT) on demineralized enamel.

**Materials and Methods:**

Sixty human premolar specimens were demineralized and randomly allocated into four groups ( *n* = 15): BAT-1, BAT-2, CPT, and untreated control (NT). Specimens underwent a 7-day pH cycling and were treated with respective materials twice daily. Surface microhardness was measured at baseline, post-demineralization, and posttreatment to calculate the percentage of hardness recovery (%HR) and remineralization potential (%RP). Structural and mineral changes were analyzed using scanning electron microscopy (SEM), atomic force microscopy (AFM), and polarized light microscopy (PLM).

**Statistical Analysis:**

ANOVA (analysis of variance) and Bonferroni's post-hoc tests were utilized to identify significant differences among groups (α = 0.05).

**Results:**

Significant variations in %HR and %RP were observed across groups, though BAT-1 and CPT performed comparably ( *p* > 0.05). SEM revealed that BAT-1 was superior to BAT-2 and CPT in preserving enamel architecture and promoting mineral penetration, effects that were negligible in the NT group. AFM showed more densely packed and better oriented nanohydroxyapatite crystals in both BAT groups compared with the others. Furthermore, PLM indicated that BAT-1 achieved the most substantial reduction in lesion depth.

**Conclusion:**

Although BAT-1 showed remineralization potential comparable to CPT, it was notably more effective at reducing lesion depth. Consequently, BAT-1 is a promising fluoride-free material for the noninvasive management of early carious lesions.

## Introduction

Dental caries remains a major global health concern due to its high prevalence and detrimental effect on quality of life. The destructive mechanism involves the creation of an acidic oral environment, primarily resulting from the bacterial breakdown of sugars within dental plaque. Demineralization of the enamel layer commences once the local pH drops below the established critical point of 5.5. 1 In contrast to this acidic challenge, normal physiological responses involve salivary buffering agents, specifically with calcium (Ca ^2+^ ) and phosphate (PO _4_^3−^ ) ions, which neutralize the acid. 2 As these salivary ions maintain a state where the oral environment is supersaturated compared with apatite, saliva effectively prevents further enamel damage and facilitates the reestablishment of tooth mineral through remineralization. 2 Despite the inherent protective and restorative properties of saliva against decay, these natural mechanisms often fail to prevent the formation of new carious lesions or the progression of existing ones, particularly under conditions characterized by excessive sugar intake, significant plaque buildup, or compromised salivary function. 1 2 Initial carious lesions manifest as white spot lesions, characterized by demineralization occurring beneath an intact outer enamel surface layer. 3 The process begins with the dissolution of the interprismatic mineral content of the enamel, which precedes the formation of a distinct, superficial layer characteristic of incipient caries. 3

Preventive dental strategies are thus fundamentally focused on achieving two critical objectives—the promotion of mineral repair and the suppression of demineralization—both of which center on augmenting the concentration of Ca ^2+^ and PO _4_^3–^ ions within the salivary fluid. 4 A range of strategies to manage dental caries has been introduced, many of which incorporate bioactive substances into oral care formulations. These agents facilitate remineralization primarily through ion exchange mechanisms, creating a supersaturated environment in the oral fluids that promotes the deposition of ions onto demineralized enamel. 5 This process results in the formation of amorphous calcium phosphate, which subsequently supports the development of hydroxyapatite (HA) crystals. 4 Fluoride is widely recognized as a key component in various oral care products due to its ability to prevent the loss of minerals from the surface of dental enamel. It works by adsorbing onto partially demineralized crystalline structures and attracting PO _4_^3–^ and Ca ^2+^ ions from saliva. This process facilitates the formation of fluorapatite (Ca _5_ (PO _4_ ) _3_ F), particularly within the superficial layers of early lesions. Because fluorapatite is more chemically stable and resilient against acid than HA, it provides the enamel with superior surface protection. 6 7 Excessive fluoride incorporation into the surface layer may be blocking the penetration of ions into the deeper subsurface regions of carious lesions, thereby preventing complete and effective remineralization. 8 Additionally, growing apprehensions regarding fluoride toxicity, combined with increased consumer preference for biocompatible and naturally derived products, have stimulated research into fluoride-free remineralizing agents. 9 The integration of casein phosphopeptide-amorphous calcium phosphate (CPP-ACP) has gained prominence as a calcium phosphate–based remineralizing agent. While formulated alongside fluoride (CPP-ACFP), the complex demonstrates enhanced efficacy in the biomimetic remineralization of incipient lesions. 10 Nevertheless, clinicians must exercise caution when prescribing CPP-based agents to patients with confirmed milk protein allergies or severe lactose sensitivities. 11 Recently, fluoride-containing functionalized tricalcium phosphate in an aqueous dentifrice base was introduced as Clinpro tooth crème (3M, St. Paul, Minnesota, United States), reported for effective tooth remineralization. 12 13 14

Among several remineralizing agents, HA (Ca _10_ (PO _4_ ) _6_ (OH) _2_ ) has gained considerable attention as a biomimetic active ingredient, which possesses the structure and composition mimicking the natural enamel crystalline structure, biocompatibility, and osteoconductive properties. 15 Nanohydroxyapatite (nHA), characterized by particulate dimensions between 20 and 100 nm, functions as a biomimetic calcium phosphate derivative that mirrors the crystallographic habit of human dental enamel. This morphological similarity facilitates deep penetration into subsurface demineralized zones. By chemically bonding with indigenous apatite, nHA precipitates a uniform restorative layer that reinstates the mechanical properties—specifically, hardness and elasticity—of the original tooth structure. 16 The porous architecture of incipient caries facilitates the infiltration of nHA, which functions as a crystalline scaffold. By sequestering calcium and phosphate ions, it promotes sustained mineral accretion and intrinsic remineralization. 17 18 19 Beyond its role as a mineral reservoir that maintains salivary supersaturation, 4 20 nHA exhibits antimicrobial efficacy by adsorbing to pathogenic biofilms. This adhesion prevents bacterial colonization and mitigates plaque accumulation, reinforcing its function as a multifaceted prophylactic agent. 21 22 Comparative analyses indicate that nHA-integrated dentifrices demonstrate superior remineralization potential (RP) relative to conventional fluoride-based alternatives. 21 22 Furthermore, the clinical utility of nHA extends to the treatment of dentinal hypersensitivity; research confirms its ability to provide symptomatic relief by effectively sealing exposed dentinal tubules. 16 23

The prevalence of caries also depends on the amount of bacteria in the oral cavity, especially *Streptococcus mutans* . Several studies reported that α-mangostin and silver from Apacider-AW are capable of reducing microorganisms in the oral cavity. 24 25 Alpha-mangostin is a xanthone, a natural extract from *Garcinia mangostana* that exhibits antimicrobial, antioxidant, and anti-inflammatory properties and induces apoptosis of cancer cells. 24 Apacider, an inorganic tricalcium phosphate carrier with silver nanoparticles, promotes the remineralization of white spot lesions while exhibiting broad-spectrum antibacterial activity. 24 25 The studies were reported on the apacider mangosteen adhesive paste (AMAP), comprising apacider, a calcium phosphate-based remineralizing agent containing silver and zinc, and α-mangostin, an antibacterial compound, for managing early carious lesions. 24 25 The study has shown that AMAP significantly increases enamel hardness and remineralization, comparable to fluoride varnish and CPP-ACP. 25 It also enhances acid resistance and helps maintain mineral gain during acid exposure.

Currently, an array of oral hygiene vehicles incorporating bioactive agents has emerged, specifically engineered to augment caries prevention and remineralization. 17 21 25 26 This proliferation of diverse formulations provides clinicians with expanded therapeutic options but simultaneously challenges the selection process. The inherent variability in these bioactive compositions may significantly affect their clinical performance regarding their efficacy in caries prevention and remineralization. To date, no research has documented the application of nHA in a toner format following routine toothbrushing. Accordingly, a novel fluoride-free calcium phosphate bioactive toner (BAT; Dent-Pharm Khon Kaen University [KKU], Thailand) was developed to provide dual antibacterial and remineralizing properties. This study evaluates two formulations: BAT-1, which incorporates nHA (Sangi, Japan), apacider, and α-mangostin; and BAT-2, which contains apacider and α-mangostin but lacks the nHA component. Due to their low viscosity relative to standard nanogels, these toners facilitate the deep infiltration of nano-sized active ingredients into enamel interprismatic spaces. This study evaluated remineralization efficacy of fluoride-free BAT-1 and BAT-2 in comparison to a fluoride-containing functionalized tricalcium phosphate toothpaste (CPT; Clinpro, 3M). The null hypothesis posits that there are no significant differences in remineralization or penetration depth between the three groups (BAT-1, BAT-2, and CPT) when compared with untreated demineralized enamel.

## Materials and Methods

Ethical approval for this investigation was granted by the KKU Ethics Committee for Human Research (Ref. No.: HE 682071), with the methodology adhering to Check list for Reporting In-vitro Studies (CRIS) guidelines for *in vitro* research. The necessary sample size was determined using PI-FACE software V-1.76 (Iowa University, Iowa, United States) based on effect sizes reported in a previous study. 25 Calculations were executed according to Eq. 1 with α = 0.05 and a statistical power of 0.90.



where Z _α =_ normal standard deviation = 1.96 (α = 0.05), Z _β =_ normal standard deviation = 1.28 (β = 0.1), µ _1_ − µ _2_ = difference of mean between groups = 5, and *s* = standard deviation ( *s*_1_ = 4.6, *s*_2_ = 3.7).

### Specimen Preparation

For this study, human premolars extracted for orthodontic purposes were collected. Criteria for inclusion required the absence of dental caries, fluorosis, developmental defects, enamel hypoplasia, or any visible fractures and lesions. Both patients and their legal guardians provided informed consent prior to the extractions. Until use, the specimens were stored in an opaque container filled with 0.1% thymol solution (M-Dent, Bangkok, Thailand). Before processing, all teeth were meticulously cleaned of soft tissue, plaque, and calculus, then rinsed with deionized (DI) water.

**Fig. 1:**
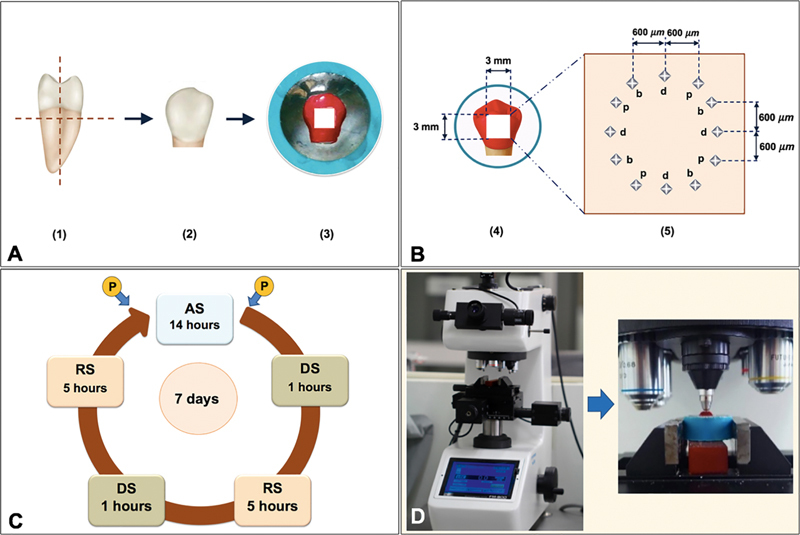
( **A** ) A human bicuspid was sectioned vertically and horizontally (1) to isolate the buccal half of the crown (2), followed by embedding in acrylic resin while leaving the buccal enamel surface exposed (3). ( **B** ) Nail varnish was applied to the enamel, leaving a 3 × 3 mm central window uncoated (4) for microhardness testing (5). Surface microhardness was measured at baseline (Hb), after demineralization (Hd), and following a 7-day pH-cycling protocol (Hp). ( **C** ) The pH-cycling process involved sequential immersion in demineralizing solution (DS), remineralizing solution (RS), and artificial saliva (AS). Application of remineralizing products (P) was performed before and after immersion in AS. ( **D** ) All measurements were performed using a Vickers diamond indenter within a microhardness testing apparatus.

To prepare the samples, a precision sectioning machine (Mecatome-T180, Presi, France) with a diamond blade and water cooling was used to divide each tooth longitudinally through the mesio-distal plane and horizontally 1 mm beneath the cervical line to segregate the buccal aspect from the lingual aspect of the crown ( Fig. 1A[1] ). The buccal crown segments ( Fig. 1A[2] ) were secured in acrylic resin, ensuring the enamel surface remained exposed above the resin interface. These surfaces were then leveled using a grinding and polishing apparatus (Ecomet, Buehler, Illinois, United States) with aluminum oxide abrasive papers up to 3,000 grit. This process created a standardized 3 × 3 mm experimental window in the center of each specimen ( Fig. 1A[3] ). To isolate the study area, all remaining enamel was sealed with nail varnish (Revlon, New York, United States), leaving only the designated test site exposed. A total of 60 specimens were prepared and subsequently immersed in DI water at 37°C. Baseline microhardness (Hb) was then measured on the enamel to provide a starting value for comparison ( Fig. 1B[4, 5] ).

**Fig. 2:**
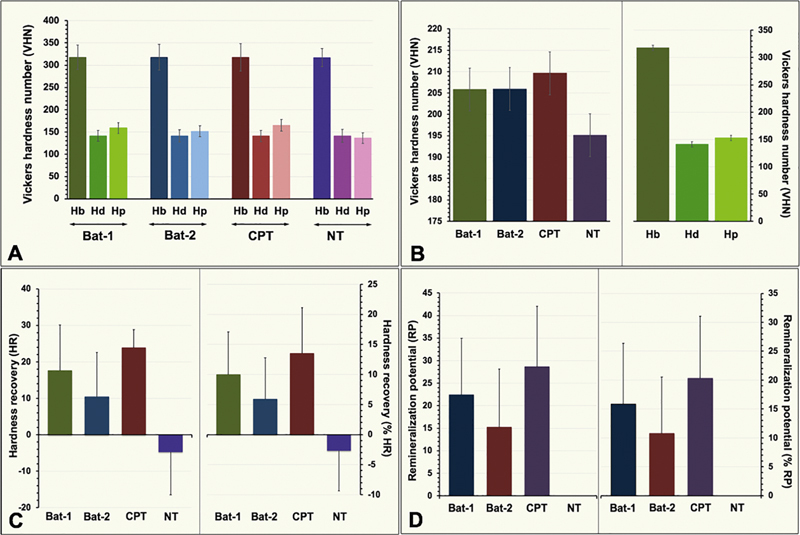
( **A** ) Mean and standard deviation values for baseline microhardness (Hb), post-demineralization hardness (Hd), and hardness following pH-cycling/remineralization (Hp). ( **B** ) Microhardness values were recorded after exposure to test materials over specified periods. ( **C** ) Absolute hardness recovery (HR) and percentage hardness recovery (%HR) are calculated after the pH-cycling and remineralization phases. ( **D** ) Remineralization potential (RP) and percentage remineralization potential (%RP) for bioactive toner formula 1 (BAT-1), bioactive toner formula 2 (BAT-2), Clinpro Tooth Crème (CPT), and the no treatment (NT) control group.

**Fig. 3:**
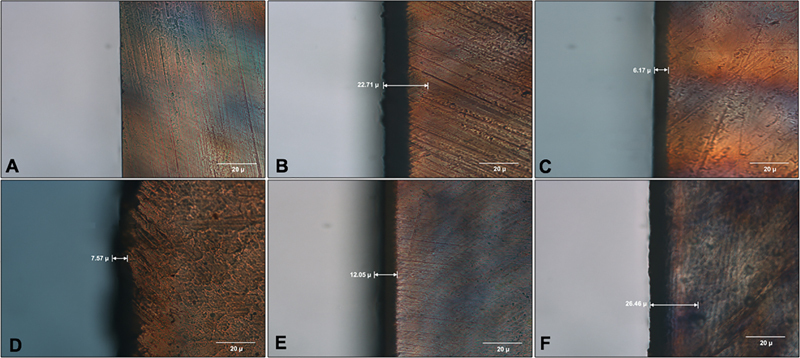
Polarized light micrographs (PLMs) at 50× magnification of ( **A** ) sound enamel, ( **B** ) enamel after artificial demineralization, and demineralized enamel treated with ( **C** ) bioactive toner formula 1 (BAT-1), ( **D** ) bioactive toner formula 2 (BAT-2), and ( **E** ) Clinpro Tooth Crème (CPT), compared with ( **F** ) an untreated control (NT).

**Fig. 4:**
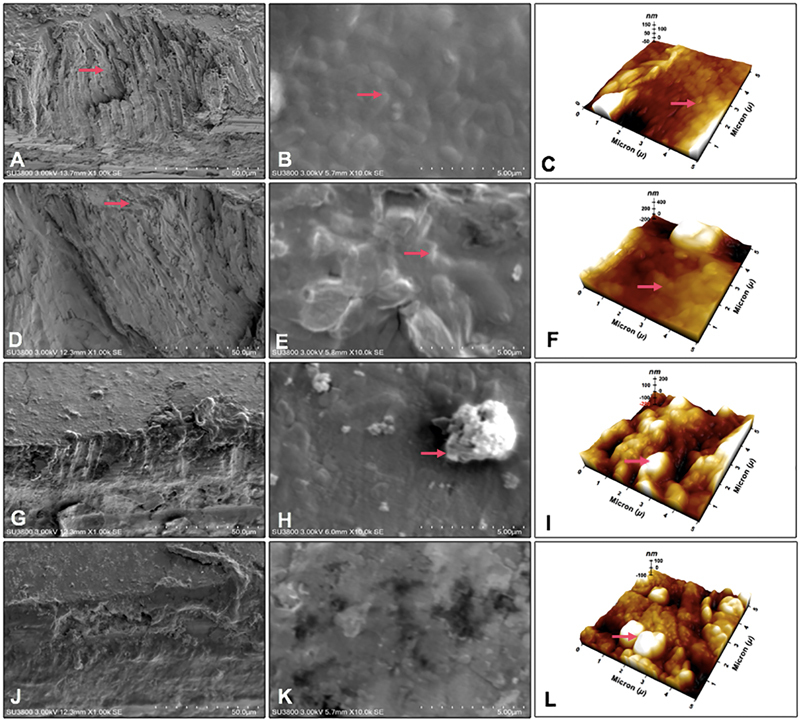
Scanning electron microscopy (SEM) cross-sectional images at 1,000× ( **A, D, G, J** ) and surface topography images at 10,000× ( **B, E, H, K** ), together with atomic force microscopy (AFM) images ( **C, F, I, L** ) of demineralized enamel after treatment with bioactive toner formula 1 (A–C), bioactive toner formula 2 (D–F), Clinpro Tooth Creme (G–I), and no treatment (NT; **J–L** ). Red arrows indicated the mineral depositions.

### Induction of Artificial Demineralized Enamel Lesion

To create a standardized subsurface artificial demineralized lesion on the enamel, an artificial caries-inducing (CI) gel was formulated. 4 This mixture consisted of 0.1 mol/L lactic acid (C _3_ H _6_ O _3_ ), 500 mg/L HA (Ca _10_ (PO _4_ ) _6_ (OH) _2_ ), and 20 g/L Carbopol-970 (BF-Goodrich, Ohio, United States), with the pH precisely adjusted to 5.0 using 1 M sodium hydroxide (NaOH). Each enamel specimen was submerged in a sterile container of CI gel and incubated for 12 hours at 37°C within a humidified chamber. After this period, the samples were rinsed with DI water to remove the gel, and the baseline demineralized surface microhardness (Hd) was recorded.

### Application of the Acid-Challenging Process and Bioactive Material

Following the initial assessment of Hd, specimens were distributed into four experimental cohorts ( *n* = 15) using a randomized sampling technique. This ensured baseline consistency before the samples were subjected to a pH-cycling acid challenge and remineralization protocol. The treatment groups were defined as follows:

Group BAT-1: application of fluoride-free BAT formula-1 (Dent-Pharm KKU, Thailand).

Group BAT-2: application of fluoride-free BAT formula-2 (Dent-Pharm KKU, Thailand).

Group CPT: application of fluoride-containing functionalized CPT (Clinpro, 3M, United States).

Group NT: a negative control group maintained in DI water without treatment.

The experimental protocol involved an acid-challenge model using demineralizing (DS), remineralizing (RS), and artificial saliva (AS) solutions, all of which were freshly prepared for each cycle ( Table 1 ). 27 The DS was formulated with 2.2 mM calcium chloride (CaCl _2_ ), 2.2 mM potassium hydrogen phosphate (KH _2_ PO _4_ ), and 0.05 M acetic acid (CH _3_ COOH), with the pH calibrated to 5.5 using 1 M potassium hydroxide (KOH). The RS consisted of 1.5 mM calcium chloride (CaCl _2_ ), 0.9 mM sodium dihydrogen phosphate (NaH _2_ PO _4_ ), and 0.15 M potassium chloride (KCl), adjusted to pH 7.0 with 1 M sodium hydroxide (NaOH). The AS was composed of a complex mixture of salts [0.65 g/L potassium chloride (KCL), 0.058 g/L magnesium chloride (MgCl _2_ ), 0.165 g/L calcium chloride (CaCl _2_ ), 0.804 g/L dipotassium hydrogen phosphate (K _2_ HPO _4_ ), 0.365 g/L potassium dihydrogen phosphate (KH _2_ PO _4_ )], 2 g/L sodium benzoate (C _6_ H _5_ COONa), 7.8 g/L sodium carboxymethyl cellulose (C _8_ H _15_ NaO _8_ ), 1 L with DI water, and balanced to pH 7.0 by 1 M potassium hydroxide (KOH).

**Table 1:** Materials, company, and compositions of materials and solutions used in this study

Materials	Company	Composition
Fluoride-free bioactive toner formula-1 (BAT-1)	Dent-Pharm KKU, Khon Kaen, Thailand	54.85% aqua, 20% sorbitol solution, 10.8% ethyl alcohol, 10% nanohydroxyapatite, 2% hydroxypropyl methylcellulose, 1.2% *Garcinia mangostana* peel extract, 0.88% propylene glycol, 0.1% peppermint oil, 0.1% methylparaben, 0.045% tricalcium phosphate, 0.02% propylparaben, 0.003% silica, 0.001% zinc oxide, 0.001% silver
Fluoride-free bioactive toner formula-2 (BAT-2)	Dent-Pharm KKU, Khon Kaen, Thailand	64.85% aqua, 20% sorbitol solution, 10.8% ethyl alcohol, 2% hydroxypropyl methylcellulose, 1.2% *Garcinia mangostana* peel extract, 0.88% propylene glycol, 0.1% peppermint oil, 0.1% methylparaben, 0.045% tricalcium phosphate, 0.02% propylparaben, 0.003% silica, 0.001% zinc oxide, 0.001% silver
Fluoride-containing functionalized tricalcium phosphate toothpaste (Clinpro tooth crème, CPT)	3M, St. Paul, Minnesota, United States	0.21% sodium fluoride, 0.95 mg of fluoride ion, water, sorbitol, hydrated silica, glycerin, polyethylenepolypropylene glycol, flavor, polyethylene glycol, sodium lauryl sulfate, titanium dioxide, carboxymethyl cellulose, sodium saccharin, and tri-calcium phosphate.
Artificial caries inducer (CI)	Biomaterial research, KKU Khon Kaen, Thailand	Lactic acid 0.1 mol/L, hydroxyapatite 500 mg/L, carbopol-907 20 g/L, pH adjusted to 5.0 with 1 M sodium hydroxide
Demineralizing solution (DS)	Biomaterial research, KKU Khon Kaen, Thailand	Calcium chloride 2.2 mM, potassium hydrogen phosphate 2.2 mM, acetic acid 0.05 M, pH adjusted to 5.5 with 1 M potassium hydroxide
Remineralizing solution (RS)	Biomaterial research, KKU Khon Kaen, Thailand	Calcium chloride 1.5 mM, sodium dihydrogen phosphate 0.9 mM, potassium chloride 0.15 M, pH adjusted to 7.0 with 1 M sodium hydroxide
Artificial saliva (AS)	Biomaterial research, KKU Khon Kaen, Thailand	Potassium chloride 0.65 g/L, magnesium chloride 0.058 g/L, calcium chloride 0.165 g/L, dipotassium hydrogen phosphate 0.804 g/L, potassium dihydrogen phosphate 0.365 g/L, sodium benzoate 2.0 g/L, sodium carboxymethyl cellulose 7.8 g/L, deionized water to make 1 L, pH adjusted to 7.0 with 1 M potassium hydroxide

Specimens were housed in 24-well plates, with 2 mL of solution per well, and placed in an orbital shaking incubator (Amerex, Alabama, United States) at 37°C and 50 rpm for 7 days. The daily pH-cycling schedule included two cycles of 1 hour in DS and 5 hours in RS, followed by a 12-hour immersion in AS ( Fig. 1C ). The application of remineralizing agents was performed twice daily, occurring both before and after immersion in AS ( Fig. 1C ). While groups BAT-1 and BAT-2 received 4-minute applications, the CPT group was treated for 2 minutes using a 1:3 toothpaste-to-DI water slurry, in accordance with the manufacturer's instructions. All specimens were rinsed with DI water post-application, whereas the NT control group was placed in DI water twice daily.

### Evaluation of Surface Hardness

Surface microhardness was measured at three distinct stages: at baseline (Hb), following the formation of artificial caries (Hd), and after the 7-day pH-cycling and remineralization protocol (Hp). For each specimen, four random indentations were made using a Vickers hardness tester (Future-tech, Tokyo, Japan) with a 100 g load applied for 15 seconds ( Fig. 1D ). To ensure independence of measurements, indentations were spaced 600 microns (μ) apart ( Fig. 1B[5] ). The Vickers hardness number (VHN) was derived from the diagonal lengths of the resulting imprints. Subsequently, the data were used to calculate hardness recovery (HR), percentage of surface HR (%HR), RP, and percentage of RP (%RP) using the formulas detailed in Eqs. 2 and 3 .



where *H*_p*_ refers to hardness after application of pH-cycling and remineralization to the no treatment (NT) group.

### Microscopic Determination

For structural analysis, three specimens per group were randomly sampled and prepared for polarized light microscopy (PLM; Eclipse-80i, Nikon, Kanagawa, Japan). These samples were longitudinally sectioned to 150 μm using a precision saw (Mecatome T180, PRESI) and polished with SiC abrasive paper up to 5,000 grit. After cleaning with DI water, the sections were viewed at 50× magnification to evaluate remineralization efficacy, using sound and demineralized enamel as benchmarks.

Furthermore, three randomly chosen samples from each cohort were prepared for scanning electron microscopy (SEM; S-3000N, Hitachi, Tokyo, Japan) and energy-dispersive spectroscopy (EDS; Oxford, High Wycombe, United Kingdom). After 24 hours of desiccation at room temperature, specimens were sputter-coated (K500X, Emitech, Ashford, United Kingdom) with gold–palladium (10 mA, 130 mTorr, 3 minutes). Surface topography was visualized at 10,000× magnification, while cross-sectional characteristics were observed at 1,000 × .

Finally, surface roughness was characterized using atomic force microscopy (AFM; XE-120, Park Systems, Suwon, Korea) on three additional samples. Imaging was performed over a 5 × 5 μ ^2^ area using a tetrahedral golden silicon probe with an Au-reflective coating (Nanosensors, Neuchatel, Switzerland). This specialized probe featured a 10 nm diameter cylindrical tip for high-resolution topographical mapping.

### Statistical Analysis

Statistical analyses were performed using SPSS version 28.0 (IBM Corp., Armonk, New York, United States). The normality of the data distribution was confirmed via the Kolmogorov–Smirnov test. Differences in surface microhardness across the experimental stages (Hb, Hd, and Hp) and calculated remineralization parameters (HR, %HR, RP, and %RP) were evaluated using a one-way analysis of variance (ANOVA). Significant inter-group differences were further identified using Bonferroni post-hoc multiple comparison tests, with the significance level established at α = 0.05. Qualitative assessments were conducted for the PLM, SEM, and AFM imaging data.

## Results

The mean, standard deviation, and 95% confidence interval of Hb, Hd, Hp, HR, %HR, RP, and %RP for each group are displayed in Table 2 and Fig. 2A . A two-way ANOVA was conducted to assess the significant impact of material type and intervention duration on enamel microhardness relative to the NT group. Statistical analysis indicated that material type, intervention period, and their interaction significantly affected enamel microhardness ( *p* < 0.05; Table 3 ). Post-hoc Bonferroni HSD multiple comparisons revealed that enamel treated with BAT-1, BAT-2, or CPT exhibited significantly higher VHN than enamel in the NT groups ( *p* < 0.05). Similarly, multiple comparisons demonstrated that enamel hardness at the Hb stage was significantly greater than at the Hp and Hd stages, respectively ( *p* < 0.05) ( Table 4 and Fig. 2B ). We detected no significant difference in Hb among the treated groups ( *p* > 0.05) through ANOVA and post-hoc Bonferroni HSD multiple comparisons, as detailed in Tables 3 and 4 . The mean Hd for each group decreased compared with the mean Hb ( Fig. 2A, B ). However, there was no significant difference in Hd among the tested groups ( *p* > 0.05), as shown in Tables 3 and 4 . Following the introduction of pH cycling and remineralization, the mean Hp for each group significantly increased compared with Hd ( *p* < 0.05), with the exception of the NT group ( *p* > 0.05), as illustrated in Fig. 2(A, B) . Significant differences in mean Hp were observed among the tested groups, as presented in Fig. 2A and Table 3 . Bonferroni's multiple comparisons confirmed significant differences in mean Hp among the tested groups ( *p* < 0.05), displayed in Table 4 . The CPT-treated group exhibited the highest HR, followed by the BAT-1 and BAT-2 groups, as detailed in Fig. 2C . Statistical analysis revealed significant differences in mean HR across the tested groups ( *p* < 0.05), with the exception of the CPT and BAT-1 comparison, where their mean HR values were found to be comparable ( Fig. 2C , Tables 3 and 4 ). Similarly, the CPT-treated group also demonstrated the highest %HR, preceding BAT-1 and BAT-2 ( Fig. 2C ). Significant variations in mean %HR were observed among the groups ( *p* < 0.05), though the %HR for CPT and BAT-1 were not statistically different ( Fig. 2C , Tables 3 and 4 ). Regarding RP, the CPT-treated group again showed the highest values, with BAT-1 and BAT-2 following in descending order ( Fig. 2D ). ANOVA confirmed significant differences in the mean RP among the groups ( *p* < 0.05), except for CPT and BAT-1, which had comparable RP values ( Fig. 2D , Tables 3 and 4 ). Correspondingly, the CPT group recorded the highest %RP, with BAT-1 and BAT-2 having lower values ( Fig. 2D ). Significant differences in mean %RP were noted across the groups ( *p* < 0.05), except for the CPT and BAT-1 groups, whose %RP values were comparable ( Fig. 2D , Tables 3 and 4 ). Based on remineralization parameters—HR, %HR, RP, and %RP—the application of BAT-1, BAT-2, and CPT significantly promoted the remineralization of demineralized enamel compared with the untreated (NT) group ( *p* < 0.05). While CPT exhibited a RP comparable to BAT-1, BAT-2 showed significantly lower efficacy than both CPT and BAT-1.

**Table 2:** Mean, standard deviation (SD), 95% confidential interval (CI) of baseline hardness (Hb), hardness after artificial demineralization (Hd), hardness after pH-cycling process (Hp), hardness recovery (HR), percentage of hardness recovery (%HR), remineralization potential (RP), and percentage of remineralization potential (%RP) for bioactive toner formular 1 (BAT-1), bioactive toner formular 2 (BAT-2), Clinpo tooth crème (CPT), and no treatment (NT) group

Group	Hb	Hd	Hp	HR	%HR	RP	%RP
	Mean ± SD CI (LL–UL)	Mean ± SD CI (LL–UL)	Mean ± SD CI (LL–UL)	Mean ± SD CI (LL–UL)	Mean ± SD 95%CI (LL–UL)	Mean ± SD CI (LL–UL)	Mean ± SD CI (LL–UL)
BAT-1	317.6 ± 27.4 ^a^ (310.4–324.9)	141.2 ± 12.4 ^b^ (137.7–144.8)	158.8 ± 12.6 ^e^ (155.5–162.2)	17.6 ± 12.6 ^g^ (14.2–20.9)	10.0 ± 7.1 ^k^ (8.1–11.9)	22.4 ± 12.6 ^o^ (19.0–25.7)	15.8 ± 8.9 ^s^ (13.7–18.2)
BAT-2	317.8 ± 29.2 ^a^ (310.9–324.8)	141.3 ± 13.6 ^b^ (137.8–144.8)	151.7 ± 12.2 ^d^ (148.4–155.0)	10.4 ± 12.2 ^h^ (7.1–13.7)	5.9 ± 6.9 ^m^ (4.0–7.8)	15.2 ± 12.9 ^p^ (11.9–18.5)	10.8 ± 8.6 ^t^ (8.4–13.1)
CPT	317.6 ± 30.7 ^a^ (310.7–324.6)	141.2 ± 12.7 ^b^ (137.8–144.7)	165.1 ± 13.4 ^c^ (161.8–168.4)	23.9 ± 13.4 ^g^ (20.6–27.2)	13.5 ± 7.6 ^k^ (11.6–15.4)	28.6 ± 13.4 ^o^ (25.3–31.9)	20.3 ± 9.5 ^s^ (17.9–22.6)
NT	317.1 ± 19.9 ^a^ (309.6–324.4)	141.2 ± 14.6 ^b^ (137.8–144.6)	136.5 ± 11.8 ^f^ (133.2–139.2)	− 4.7 ± 11.7 ^j^ [(−7.9)–(−1.5)]	− 2.7 ± 6.7 ^n^ [(−4.5)–(−0.9)]	0.0 ± 11.8 ^r^ [(−3.2)–3.2]	0.0 ± 8.3 ^v^ [(−1.3)–2.3)]

Abbreviations: LL, lower limit; UL, upper limit.

Note: Different superscript letters in the same column represent a significant difference between treatment groups ( *p* < 0.05).

**Table 3:** An analysis of variance (ANOVA) of hardness alteration upon treatment with different materials at different periods, baseline hardness (Hb), hardness after artificial demineralization (Hd), hardness after pH-cycling process (Hp), hardness recovery (HR), percentage of hardness recovery (%HR), remineralization potential (RP), and percentage of remineralization potential (%RP)

**a. Two-way ANOVA of hardness alteration upon treatment with different materials at different periods**					
**Source**	**SS**	**df**	**MS**	***F***	***p***
Intercept	28,243,363.990	1	28,243,363.990	78,260.168	0.001
Materials	9,076.452	3	3,025.484	8.383	0.001
Period	4,415,822.566	2	2,207,911.283	6,117.951	0.001
Materials * periods	16,934.013	6	2,822.335	7.820	0.001
Error	241,074.964	668	360.891		
Total	33,029,259.594	680			
**b. One-way ANOVA of baseline hardness (Hb)**					
**Source**	**SS**	**df**	**MS**	***F***	***p***
Between group	19.550	3	6.517	0.009	0.999
Within group	166,609.902	224	743.794		
Total	23,159,478.688	228			
**c. One-way ANOVA of after demineralization (Hd)**					
**Source**	**SS**	**df**	**MS**	***F***	***p***
Between group	0.268	3	0.089	0.000	1.000
Within group	40,560.202	226	179.470		
Total	4,628,713.622	230			
**d. One-way ANOVA of after application of pH-cycling and remineralization process (Hp)**					
**Source**	**SS**	**df**	**MS**	***F***	***p***
Between group	103,955.505	3	34,651.835	2,738.627	0.001
Within group	961.628	76	12.653		
Total	1,945,596.709	80			
**e. One-way ANOVA of hardness recovery (HR)**					
**Source**	**SS**	**df**	**MS**	***F***	***p***
Between group	25,769.477	3	8,589.826	55.230	0.001
Within group	33,904.860	218	155.527		
Total	89,199.671	222			
**f. One-way ANOVA of percentage of hardness recovery (%HR)**					
**Source**	**SS**	**df**	**MS**	***F***	***p***
Between group	8,289.053	3	2,763.018	55.227	0.001
Within group	10,906.666	218	50.031		
Total	28,672.711	222			
**g. One-way ANOVA of remineralization potential (RP)**					
**Source**	**SS**	**df**	**MS**	***F***	***p***
Between group	25,857.879	3	8,619.293	55.420	0.001
Within group	33,904.860	218	155.527		
Total	118,831.036	222			
**h. One-way ANOVA of percentage of remineralization potential (%RP)**					
**Source**	**SS**	**df**	**MS**	***F***	***p***
Between group	12,963.895	3	4,321.298	55.429	0.001
Within group	16,995.540	218	77.961		
Total	59,565.089	222			

Abbreviations: df, degree of freedom; *F* , *F* -ratio; MS, mean square; SS, sum of squares.

**Table 4:** Post-hoc Bonferroni HSD multiple comparisons of hardness alteration upon treatment with different materials at different periods, baseline hardness (Hb), hardness after artificial demineralization (Hd), hardness after pH-cycling process (Hp), hardness recovery (HR), percentage of hardness recovery (%HR), remineralization potential (RP), and percentage of remineralization potential (%RP) among bioactive toner formular 1 (BAT-1), bioactive toner formular 2 (BAT-2), Clinpo tooth crème (CPT), and no treatment (NT) group

**a. Bonferroni HSD multiple comparison of hardness alteration upon treatment with different materials at different periods**								
**Group**	**NT**	**BAT-1**	**BAT-2**	**CPT**	**Period**	**Hb**	**Hd**	**Hp**
NT	1.000	0.001	0.001	0.001	Hb	1.000	0.001	0.001
BAT-1		1.000	1.000	0.392	Hd		1.000	0.001
BAT-2			1.000	0.436	Hp			1.000
CPT				1.000				
**b. Bonferroni HSD multiple comparison of baseline hardness (Hb)**								
**Group**	**NT**		**BAT-1**		**BAT-2**		**CPT**	
NT	1.000		1.000		1.000		1.000	
BAT-1			1.000		1.000		1.000	
BAT-2					1.000		1.000	
CPT							1.000	
**c. Bonferroni HSD multiple comparison after artificial formation of demineralization (Hd)**								
**Group**	**NT**		**BAT-1**		**BAT-2**		**CPT**	
NT	1.000		1.000		1.000		1.000	
BAT-1			1.000		1.000		1.000	
BAT-2					1.000		1.000	
CPT							1.000	
**d. Bonferroni HSD multiple comparison of after application of pH-cycling and remineralization (Hp)**								
**Group**	**NT**		**BAT-1**		**BAT-2**		**CPT**	
NT	1.000		0.001		0.001		0.001	
BAT-1			1.000		0.016		0.046	
BAT-2					1.000		0.001	
CPT							1.000	
**e. Bonferroni HSD multiple comparison of hardness recovery (HR)**								
**Group**	**NT**		**BAT-1**		**BAT-2**		**CPT**	
NT	1.000		0.001		0.001		0.001	
BAT-1			1.000		0.017		0.056	
BAT-2					1.000		0.001	
CPT							1.000	
**f. Bonferroni HSD multiple comparison of percentage of hardness recovery (%HR)**								
**Group**	**NT**		**BAT-1**		**BAT-2**		**CPT**	
NT	1.000		0.001		0.001		0.001	
BAT-1			1.000		0.017		0.056	
BAT-2					1.000		0.001	
CPT							1.000	
**g. Bonferroni HSD multiple comparison of remineralization potential (RP)**								
**Group**	**NT**		**BAT-1**		**BAT-2**		**CPT**	
NT	1.000		0.001		0.001		0.001	
BAT-1			1.000		0.019		0.056	
BAT-2					1.000		0.001	
CPT							1.000	
**h. Bonferroni HSD multiple comparison of percentage of remineralization potential (%RP)**								
**Group**	**NT**		**BAT-1**		**BAT-2**		**CPT**	
NT	1.000		0.001		0.001		0.001	
BAT-1			1.000		0.018		0.056	
BAT-2					1.000		0.001	
CPT							0.001	

PLM was employed to characterize the progression of enamel remineralization across the BAT-1, BAT-2, CPT, and NT groups ( Fig. 3C–F ), using sound enamel ( Fig. 3A ) and initial demineralized enamel ( Fig. 3B ) as benchmarks. The artificial demineralization process initially produced a distinct lesion body with a depth of approximately 22.71 µm ( Fig. 3B ). Following pH-cycling and treatment with the bioactive agents, a reduction in lesion depth was observed in groups treated with bioactive materials ( Fig. 3C–E ). Conversely, the negative control (NT) group displayed an increase in lesion depth relative to the baseline ( Fig. 3F ), suggesting that remineralization did not occur without bioactive materials treatment. Among the experimental groups, BAT-1 demonstrated the most pronounced remineralization, characterized by the greatest reduction in lesion depth and a significantly thicker remineralized zone compared with BAT-2 and CPT. This superior performance was attributed to the deeper penetration of nanosized calcium phosphate into the demineralized enamel lesion, whereas the CPT group showed more superficial fluorapatite deposition ( Fig. 4 ). These results underscore the efficacy of these bioactive materials in reversing artificial enamel lesions compared with the untreated control (NT).

Enamel remineralization was characterized using SEM, EDS, and AFM ( Fig. 4 ). Cross-sectional SEM images at 1,000× magnification ( Fig. 4A, D, G, J ) and topographical surface views at 10,000× magnification ( Fig. 4B, E, H, K ) provided microscopic evidence of treatment efficacy. Unlike the NT group, the BAT-1, BAT-2, and CPT groups all exhibited mineral deposition on the demineralized surfaces. Both BAT-1 and BAT-2 treatments preserved the natural enamel architecture; however, BAT-1 demonstrated superior structural integrity ( Fig. 4A, B ) compared with BAT-2 ( Fig. 4D, E ). Micrographs of these groups revealed that mineral precipitates covered the prismatic and interprismatic structures ( Fig. 4B, E ) and successfully penetrated deep into the lesion body ( Fig. 4A, D ). In contrast, the CPT-treated group showed a loss of enamel architecture, with mineral deposits restricted to the superficial layers ( Fig. 4G, H ). The NT group displayed the most significant degradation, characterized by indistinguishable prism structures and high surface porosity, indicating a complete lack of remineralization following pH-cycling ( Fig. 4J, K ).

AFM analysis revealed distinct morphological variations in the remineralized surface structures across the different treatment groups ( Fig. 4C, F, I, L ). In the BAT-1 and BAT-2 groups, the nHA crystals were characterized as minute, densely packed, and highly organized ( Fig. 4C, F ). This morphology contrasted sharply with the fluorapatite crystals observed in the CPT group ( Fig. 4I ) and the calcium-phosphate structures in the NT group ( Fig. 4L ), both of which appeared larger and more loosely arranged.

## Discussion

In modern restorative dentistry, the remineralization of early-stage carious lesions represents a key preventive strategy. This *in vitro* investigation evaluated whether the twice-daily application of BAT-1 and BAT-2 could successfully facilitate the remineralization of emerging enamel lesions within a pH-cycling environment. Using multidimensional analysis—including VHN, PLM, SEM, and AFM—the results demonstrated that BAT-1, BAT-2, and CPT all significantly enhanced remineralization compared with the NT. Consequently, the null hypothesis regarding remineralization efficacy was rejected, as the experimental interventions clearly outperformed the nontreated samples. Notably, the remineralizing potential of the BATs appeared to be on par with that of the functionalized CPT.

Artificial enamel demineralization can be achieved through various established methodologies. 10 25 26 To accurately simulate natural caries, these methods must create subsurface lesions while maintaining a relatively intact superficial layer. While some researchers employ lactate or acetate gels (pH 4.4–5.0) to mimic the organic acids produced by cariogenic bacteria, 3 this study utilized a synthetic polymer gel as a CI. This CI was composed of lactic acid, HA, and polyacrylic acid (Carbopol-907), with the latter serving as a critical stabilizing agent to preserve the surface layer during subsurface lesion formation. 4 To replicate the dynamic mineral exchange found in the oral cavity, a 7-day pH-cycling model was implemented. This model alternates between acidic challenges and mineral recovery, reflecting the natural fluctuations of mineral loss and gain. While this *in vitro* design lacks a microbial biofilm, it effectively simulates the pH swings caused by bacterial acid production and the subsequent buffering action of saliva. A demineralizing solution (pH 5.5) was specifically chosen to initiate early carious lesions, as this value represents the critical pH threshold for enamel dissolution. Following a protocol modeled after previous 7-day studies on nHA pastes, remineralizing agents were applied according to manufacturer guidelines, while the negative control (NT) remained exposed only to the demineralizing environment. 9 28

The partial remineralization of artificial enamel lesions observed in this research may be linked to an insufficient supply of calcium and phosphate ions. This ionic deficit is likely a consequence of HA crystal dissolution during the initial formation of the carious lesions, which subsequently hinders the synthesis of fluorapatite and calcium fluoride. 5 8 The BAT-1 is mainly composed of nHA and calcium phosphate from apacider, without fluoride, while the BAT-2 mainly comprises calcium phosphate-based apacider without nHA or fluoride, which closely resembles the apatite crystals found in tooth enamel, allowing them to serve as reservoirs of calcium and phosphate ions. 4 16 Owing to their small particle size, nHA and apacider particles can penetrate and directly fill the micropores within subsurface lesions. This phenomenon is similar to other studies on enamel remineralization, using carbonate–HA nanocrystals, which have observed the deposition of these crystals within eroded enamel surface scratches, forming a durable, biomimetic mineral coating. 29 30 31 32 Mineral diffusion is heavily influenced by the structural properties of artificial carious lesions, specifically their depth and porosity. While high porosity generally facilitates greater mineral accumulation, the overall rate of remineralization tends to decrease in deeper lesions because ions must travel a greater distance before deposition occurs. The superior performance of the BAT-1 group can be attributed to its nHA ions; their small size allows for deeper penetration into the enamel pores, resulting in a substantially higher level of inorganic mineral deposition compared with the BAT-2 and CPT groups. The study was supported by the cross-sectional SEM, which revealed evidence that BAT-1 exhibited a well-preserved enamel architecture, as the prism and interprism structures were not only covered with calcium phosphate depositions but also had deeper penetration of nanosized calcium phosphate into the lesion more than in BAT-2. In contrast, the CPT group exhibited a loss of enamel architecture, with strong surface rehardening via fluorapatite deposition occurring only on the surface of the lesion, with limited subsurface penetration.

Both BAT-1 and BAT-2 were formulated as topical interventions designed for noninvasive application to the tooth surface. The mineral gain process of BAT-1 is associated with nHA and apacider that function as the reservoirs for phosphate and calcium ions. These minerals can be deposited on the tooth surface and absorbed into the deeper layers of enamel porosities, resulting in a significantly higher accumulation of minerals at subsurface lesions. The mineral gain for BAT-2 is associated with calcium phosphate content in apacider and silica, which possibly has less effectiveness in remineralization than BAT-1, as supported by the microscopic evidence. CPT is composed of fluoride-containing functionalized tricalcium phosphate, which interacts at the tooth surface. This reaction enhances the bioavailability of calcium and fluoride ions and leads to the rebuilding of fluorapatite at the superficial demineralized enamel lesion. 13 This is possibly due to the larger crystal size of fluorapatite compared with nHA; its deposition is limited to the shallow surface of the lesion and the limited concentration of fluoride with calcium and phosphate in saliva; thus, the efficiency of the remineralization process could not be fully completed, as supported by other studies. 13 19 This study found that BAT-1 exhibited a higher efficiency in remineralization compared with the CPT-treated group, as demonstrated by the microscopic evidence. However, the CPT-treated groups displayed a rougher surface texture with minor porosities and loss of enamel architecture, with fluorapatite deposition limited to the shallow surface of the lesion, as reflected by the higher surface microhardness. Furthermore, the degree of remineralization is determined by the saturation level of calcium and phosphate. The AS, which contains calcium and phosphate, also demonstrated a remineralizing potential by producing a rehardening effect on the NT group.

The PLM was employed to evaluate initial lesions and differentiate enamel structures. It facilitates the accurate and straightforward quantification of demineralization depth, providing a robust framework for comparing the experimental materials. Microscopic analysis following lesion induction, but prior to treatment, revealed the dark zone located just beneath the superficial layer of the enamel. The specimen treated with BAT-1 exhibited a slightly thinner dark zone beneath the enamel surface and a thicker brown zone underneath it when compared with the BAT-2, CPT, and NT groups. This observation indicates a higher degree of remineralization for BAT-1 than for others. In contrast, the CPT-treated group showed a notably smaller brown zone than both the BAT-1 and BAT-2 groups. The analysis of the PLM micrographs revealed that the BAT-1-treated group achieved the greatest decrease in the depth of demineralized enamel compared with the other groups. This superior performance is likely attributed to BAT-1's component of nHA particles that synergistically effect remineralization with apacider, resulting in higher effectiveness in remineralization than BAT-2. Since the nHA particles are smaller than the fluorapatite crystals formed by the CPT-treated group, this grants BAT-1 its superior efficacy in remineralization capability compared with others. Due to their nanoscale size, these crystals effectively penetrate and accumulate within the pores of a carious lesion, ultimately reducing its overall depth.

While the quantitative data for %HR and %RP suggested that BAT-1 and CPT performed similarly, qualitative analysis through PLM and SEM revealed that BAT-1 actually possessed superior remineralizing capabilities. This enhanced performance is likely due to the ability of BAT-1's HA nanocrystals to form a dense, uniform mineral layer that fully masks the underlying enamel lesions. This deposition is driven by the nanocrystals' high bioactivity and their structural and chemical resemblance to natural enamel, a mechanism well-documented in the existing literature. 19 26 28 Furthermore, this study demonstrated that both BAT-1 and CPT contributed to enhanced surface hardness following pH-cycling. While CPT recorded slightly higher hardness values, the difference between the two treatments lacked statistical significance. The elevated surface hardness in the CPT group is likely attributable to the reaction between fluoride and functionalized tri-calcium phosphate. This interaction generates large fluorapatite crystals that obstruct surface pores, thereby restricting the diffusion of fluoride into the deeper layers of the lesion. 14 19 Such a mechanism explains why CPT exhibited significant surface HR but showed a less substantial reduction in demineralization depth compared with BAT-1, as seen in the PLM and SEM cross-sectional images.

The limitation of this study is subject to several constraints typical of *in vitro* research. The pH-cycling model utilized here remains a simplified version of the oral cavity, as it does not account for the protective salivary pellicle or the presence of microbial biofilms. The natural heterogeneity of the tooth samples—stemming from different donor ages and historical oral conditions—likely influenced how each specimen responded to the acidic challenges. The experimental durations for demineralization and remineralization were shorter than those typically observed in a biological setting. Additionally, the limited use of quantitative mineral analysis beyond microhardness measurement was performed. Therefore, prospective *in situ* and clinical trials are required to confirm these observations. Nevertheless, the research offers substantial clinical value by advancing the development of novel bioactive remineralizing agents and providing a foundation for evidence-based dental practices.

## Conclusion

This *in vitro* study demonstrated that two fluoride-free BATs—BAT-1 (nHA + calcium phosphate + antibacterial agents) and BAT-2 (calcium phosphate + antibacterial agents)—along with a fluoride-containing functionalized CPT were all effective in reversing initial carious lesions. While BAT-1 and CPT exhibited comparable RP regarding surface microhardness, BAT-1 was uniquely superior in reducing the actual depth of the enamel lesion compared with all other groups. Consequently, BAT-1 appears to be a promising calcium phosphate–based, fluoride-free alternative for the noninvasive management of initial carious lesions. Further *in vivo* research is warranted to validate the clinical efficacy and cost-effectiveness of these BATs, thereby offering a clearly advantageous, economical alternative for dental therapy. Their application holds significant potential for improving oral health outcomes, particularly in high-caries-risk populations who may face challenges in maintaining conventional oral hygiene.

## Clinical Implication

Both fluoride-free BAT formulations demonstrated significant efficacy in remineralizing enamel. While BAT-1 and CPT exhibited comparable RP, the superior ability of BAT-1 to reduce lesions suggests it may provide more robust protection against lesion progression. Consequently, the synergistic combination of nHA, calcium phosphate, and antibacterial agents in BAT-1 offers a promising, noninvasive therapeutic strategy for early-stage caries. This material serves as an effective alternative for patients seeking fluoride-free treatments or those at high risk for caries. Longitudinal clinical trials are necessary to confirm these results in a complex oral environment.

## References

[1] Meyer F Enax J Epple M Amaechi B T Simader B Cariogenic biofilms: development, properties, and biomimetic preventive agents Dent J 2021 9 08 88 10.3390/dj9080088 PMC8394942 34436000

[2] Lynge Pedersen A M Belstrøm D The role of natural salivary defences in maintaining a healthy oral microbiota J Dent 2019 80 01 S3 S12 30696553 10.1016/j.jdent.2018.08.010

[3] Seevalingam R Yahaya N Syed Mohamed A MF Kumar H A Development of an artificial white spot lesion creation protocol: a preliminary study Cureus 2024 16 05 e60226 38868265 10.7759/cureus.60226 PMC11168806

[4] Cochrane N J Shen P Yuan Y Reynolds E C Ion release from calcium and fluoride containing dental varnishes Aust Dent J 2014 59 01 100 105 24494654 10.1111/adj.12144

[5] Siew B Enax J Meyer F Case report on caries assessment using intraoral scanner compared with bitewing radiographs Eur J Dent 2024 18 03 957 962 38698612 10.1055/s-0044-1782192 PMC11290935

[6] Simmer J P Hardy N C Chinoy A F Bartlett J D Hu J C How fluoride protects dental enamel from demineralization J Int Soc Prev Community Dent 2020 10 02 134 141 32670900 10.4103/jispcd.JISPCD_406_19 PMC7339990

[7] Saad H Escoube R Babajko S Houari S Fluoride intake through dental care products: a systematic review Front Oral Health 2022 3 916372 35757442 10.3389/froh.2022.916372 PMC9231728

[8] Kohli N Hugar S M Hallikerimath S Gokhale N Kadam K Soneta S P Comparative evaluation of antibacterial efficacy and remineralization potential of acidulated phosphate fluoride gel with herbal dental gel containing zingiber officinale, salvadora persica, and cinnamomum zeylanicum: an in vitro study Int J Clin Pediatr Dent 2024 17 03 307 315 39144523 10.5005/jp-journals-10005-2796 PMC11320803

[9] Buzalaf M A Hannas A R Magalhães A C Rios D Honório H M Delbem A C pH-cycling models for in vitro evaluation of the efficacy of fluoridated dentifrices for caries control: strengths and limitations J Appl Oral Sci 2010 18 04 316 334 20835565 10.1590/S1678-77572010000400002 PMC5349073

[10] Kumar V L Itthagarun A King N M The effect of casein phosphopeptide-amorphous calcium phosphate on remineralization of artificial caries-like lesions: an in vitro study Aust Dent J 2008 53 01 34 40 18304239 10.1111/j.1834-7819.2007.00006.x

[11] Xu J Shi H Luo J Advanced materials for enamel remineralization Front Bioeng Biotechnol 2022 10 985881 36177189 10.3389/fbioe.2022.985881 PMC9513249

[12] Aziz S Loch C Li K C Anthonappa R Meldrum A Ekambaram M Remineralization potential of dentifrices with calcium sodium phosphosilicate and functionalized tri-calcium phosphate in the deeper incipient carious lesions: an in vitro study Clin Exp Dent Res 2024 10 02 e876 38506322 10.1002/cre2.876 PMC10952118

[13] Barrera-Ortega C C Rodil S E Silva-Bermudez P Delgado-Cardona A Almaguer-Flores A Prado-Prone G Fluoride casein phosphopeptide and tri-calcium phosphate treatments for enamel remineralization: effects on surface properties and biofilm resistance Dent J 2025 13 06 246 10.3390/dj13060246 PMC12192112 40559149

[14] Buckshey S Anthonappa R P King N M Itthagarun A Remineralizing potential of Clinpro ^(®)^ and Tooth Mousse Plus ^®^ on artificial carious lesions J Clin Pediatr Dent 2019 43 02 103 108 30730799 10.17796/1053-4625-43.2.6

[15] Pepla E Besharat L K Palaia G Tenore G Migliau G Nano-hydroxyapatite and its applications in preventive, restorative and regenerative dentistry: a review of literature Ann Stomatol (Roma) 2014 5 03 108 114 25506416 PMC4252862

[16] Pawinska M Paszynska E Limeback H Hydroxyapatite as an active ingredient in oral care: an international symposium report Bioinspired Biomimet Nanobiomat 2024 13 01 1 14

[17] Amaechi B T Tan A I Noureldin A AK In vitro evaluation of the ability of nanohydroxyapatite toothpastes to enhance remineralization of enamel caries lesion J Dent 2025 161 106006 40744295 10.1016/j.jdent.2025.106006

[18] Anil A Ibraheem W I Meshni A A Preethanath R S Anil S Nano-Hydroxyapatite (nHAp) in the remineralization of early dental caries: a scoping review Int J Environ Res Public Health 2022 19 09 5629 35565022 10.3390/ijerph19095629 PMC9102186

[19] Huang S Gao S Cheng L Yu H Remineralization potential of nano-hydroxyapatite on initial enamel lesions: an in vitro study Caries Res 2011 45 05 460 468 21894006 10.1159/000331207

[20] Imran E Cooper P R Ratnayake J Ekambaram M Mei M L Potential beneficial effects of hydroxyapatite nanoparticles on caries lesions in vitro—a review of the literature Dent J 2023 11 02 40 10.3390/dj11020040 PMC9955150 36826185

[21] Juntavee A Juntavee N Hirunmoon P Remineralization potential of nanohydroxyapatite toothpaste compared with tricalcium phosphate and fluoride toothpaste on artificial carious lesions Int J Dent 2021 2021 5.588832E6 10.1155/2021/5588832 PMC8007336 33824661

[22] Kasemkhun P Rirattanapong P The efficacy of non-fluoridated toothpastes on artificial enamel caries in primary teeth: an in vitro study J Int Soc Prev Community Dent 2021 11 04 397 401 34430500 10.4103/jispcd.JISPCD_64_21 PMC8352058

[23] Limeback H Enax J Meyer F Clinical evidence of biomimetic hydroxyapatite in oral care products for reducing dentin hypersensitivity: an updated systematic review and meta-analysis Biomimetics (Basel) 2023 8 01 23 36648809 10.3390/biomimetics8010023 PMC9844412

[24] Majdalawieh A F Terro T M Ahari S H Abu-Yousef I A α-Mangostin: A xanthone derivative in mangosteen with potent anti-cancer properties Biomolecules 2024 14 11 1382 39595559 10.3390/biom14111382 PMC11591772

[25] Juntavee A Juntavee N Pongpanatnukul C Kruemai K Limrachtamorn T Remineralization potential of apacider mangosteen adhesive pastes on artificial carious lesions J Dent Sci 2024 19 02 978 989 38618135 10.1016/j.jds.2023.07.012 PMC11010799

[26] Juntavee A Juntavee N Sinagpulo A N Nano-hydroxyapatite gel and its effects on remineralization of artificial carious lesions Int J Dent 2021 2021 7.256056E6 10.1155/2021/7256056 PMC8592696 34790238

[27] ten Cate J M Duijsters P PE Alternating demineralization and remineralization of artificial enamel lesions Caries Res 1982 16 03 201 210 6953998 10.1159/000260599

[28] Daas I Badr S Osman E Comparison between fluoride and nano-hydroxyapatite in remineralizing initial enamel lesion: an in vitro study J Contemp Dent Pract 2018 19 03 306 312 29603704

[29] Lelli M Putignano A Marchetti M Remineralization and repair of enamel surface by biomimetic Zn-carbonate hydroxyapatite containing toothpaste: a comparative in vivo study Front Physiol 2014 5 333 25249980 10.3389/fphys.2014.00333 PMC4155874

[30] Bossù M Saccucci M Salucci A Enamel remineralization and repair results of Biomimetic Hydroxyapatite toothpaste on deciduous teeth: an effective option to fluoride toothpaste J Nanobiotechnology 2019 17 01 17 30683113 10.1186/s12951-019-0454-6 PMC6346538

[31] Limeback H Enax J Meyer F Biomimetic hydroxyapatite and caries prevention: a systematic review and meta-analysis Can J Dent Hyg 2021 55 03 148 159 34925515 PMC8641555

[32] O'Hagan-Wong K Enax J Meyer F Ganss B The use of hydroxyapatite toothpaste to prevent dental caries Odontology 2022 110 02 223 230 34807345 10.1007/s10266-021-00675-4 PMC8930857

